# Comparative analysis of curative effect of bone marrow mesenchymal stem cell and bone marrow mononuclear cell transplantation for spastic cerebral palsy

**DOI:** 10.1186/s12967-017-1149-0

**Published:** 2017-02-24

**Authors:** Xuebin Liu, Xiaojun Fu, Guanghui Dai, Xiaodong Wang, Zan Zhang, Hongbin Cheng, Pei Zheng, Yihua An

**Affiliations:** Department of Cell Transplantation, General Hospital of Chinese people’s Armed Police Forces, Beijing, 100039 China

**Keywords:** Spastic cerebral palsy, Bone marrow mesenchymal stem cells, Bone marrow mononuclear cells

## Abstract

**Background:**

Bone marrow mesenchymal stem cells (BMMSCs) and bone marrow mononuclear cells (BMMNCs) are both used to treat spastic cerebral palsy. However, the differences in therapeutic effect remain unknown.

**Methods:**

A total of 105 patients with spastic cerebral palsy were enrolled and randomly assigned to three groups: the BMMSC group, the BMMNC group and the control group. Patients in both transplantation groups received four intrathecal cell injections. Patients in the control group received Bobath therapy. The gross motor function measure (GMFM) and the fine motor function measure (FMFM) were used to evaluate the therapeutic efficacy before transplantation and 3, 6, and 12 months after transplantation.

**Results:**

Three months after cell transplantation, scores in the A dimension of GMFM and the A and C dimensions of FMFM scores in the BMMSC group are all higher than those of the BMMNC and the control groups (P < 0.05). Six months after cell transplantation, scores in the A, B dimensions of GMFM and the A, B, C, D, and E dimensions of FMFM scores in the BMMSC group are higher than those of the BMMNC and the control groups (P < 0.05). Twelve months after cell transplantation, scores in the A, B, and C dimensions of GMFM and the A, B, C, D, and E dimensions of FMFM scores in the BMMSC group are all higher than those of the BMMNC and the control groups (P < 0.05). No obvious adverse effects were investigated during follow-up.

**Conclusions:**

BMMSC transplantation for the treatment of cerebral palsy is safe and feasible, and can improve gross motor and fine motor function significantly. In addition, compared with BMMNC, the motor function of children improved significantly in terms of gross motor and fine motor functions.

**Electronic supplementary material:**

The online version of this article (doi:10.1186/s12967-017-1149-0) contains supplementary material, which is available to authorized users.

## Background

Spastic cerebral palsy (CP) is a type of non-progressive brain disorder resulting from various brain injuries that occurred in the period from conception to 1 month after childbirth. Its main clinical manifestations are motor dysfunction, abnormal posture, and, often, blindness, deafness, epilepsy, mental retardation, and other symptoms. The causes of spastic CP include periventricular leukomalacia, cerebral dysplasia, hypoxia and intrapartum asphyxia, intracranial haemorrhage and multiple other factors [1]. According to statistics, 1.5–2.5 children per 1000 of the population in developed countries, have spastic CP, and this can be even higher in developing countries [2]. Such a high incidence has placed a heavy burden on families and society.

At present, the treatment of children with spastic CP is limited to traditional methods, including physical therapy, rehabilitation training, language training, orthopaedic surgery, denervation, and intramuscular injection of botulinum toxin and other symptomatic treatment, but the effects are unsatisfactory [3]. In recent years, cell transplantation in the treatment of spastic CP has resulted in positive effects in both animal experiments and clinical studies [4–7]. Among the many kinds of stem cells, bone marrow mesenchymal stem cells (BM-MSCs) have become a popular form of seed cell transplantation because of their convenience, low immunogenicity, and amplification ability [8]. Our previous clinical experiments prove that BMMSC transplantation can effectively improve the symptoms of spastic CP, including motor function, language and cognition [9–11]. Recently, research has also confirmed that treatment with bone marrow mononuclear cell (BMMNC) transplantation has also had positive and significant effects for spastic CP [4]. These encouraging results indicate that cell transplantation for the treatment of spastic CP has broad prospects in the field. However, whether BMMSC and BMMNC from bone marrow have any difference of curative effect for the treatment of spastic CP remains unreported. Therefore, to understand whether there are any significant differences between BMMSC and BMMNC transplantation, and between BMMSC and BMMNC and traditional rehabilitation treatment for children with spastic CP, we designed this experiment.

## Methods

### Study design

**Fig. 1:**
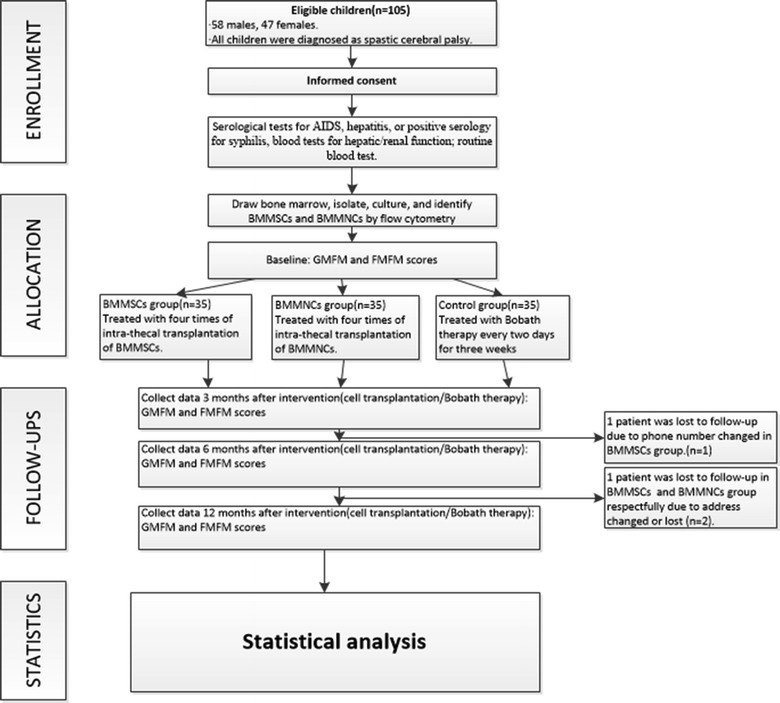
Experimental design

This study is a prospective, randomised, parallel group study. The study was approved by the General Hospital of Chinese People’s Armed Police Forces Medical Ethics Committee and has been registered in WHO (registration number CHiCTR-TRC-12002568). The patients’ families signed informed consent and understood the purpose and the significance of this study, the possible benefits and the risks of side effects (e.g., pain, fever, infection, worsening of motor function and other unpredictable side effects and the corresponding remedies). Figure 1 summarises the experimental design.

### Patients

This study involved 105 CP patients who were enrolled from May 1, 2010, to October 31, 2012. Patients were randomly assigned into the BMMSC group, the BMMNC group or the control group in a 1:1:1 ratio. The randomisation table was generated by SAS software. After randomisation, the study processes were blinded to the patients in the BMMSC and BMMNC groups, participant surgeons, coordinators, and the investigators who were responsible for patient assessment.

The case inclusion criteria were as follows: (1) children 6 to 150 months of age diagnosed with spastic CP; (2) gross motor function classification system (GMFCS) score between levels II and V; (3) no interferences due to other related treatments within 3 months prior to the enrolment and during the treatment, such as rehabilitation, traditional Chinese medicine, and surgery; and (4) parents voluntarily accepted UCMSC transplantation therapy and agreed to cooperate with follow-up studies. The case exclusion criteria were as follows: (1) patients with a history of severe allergic or autoimmune disease; (2) patients with a history of intractable seizures; (3) patients with AIDS, hepatitis, or positive serology for syphilis; (4) patients with hereditary metabolic diseases of the nervous system; (5) patients with tumours and/or blood disease history; and (6) patients who were rejected due to other serious diseases, such as brain tumours or mental and psychological disorders. All patients were placed randomly into three groups. The BMMSC group consisted of 19 boys and 16 girls with a mean age of 49.55 ± 31.95 months (range, 6–132 months). The BMMNC group consisted of 20 boys and 15 girls with a mean age of 49.10 ± 29.39 months (range, 10–150 months). The control group included 35 patients with spastic CP who were treated with rehabilitation therapy. The control group consisted of 19 boys and 16 girls with a mean age of 49.26 ± 31.31 months (range, 7–140 months). The gross motor function classification system (GMFCS) was used to classify the severity of the disease, the GMFCS levels for all patients were II–V.

### Cell preparation

#### Preparation of human BMMNCs

The parents of the patients signed informed consent. Patients were taken into the operating room, and placed in the left decubitus position and with their right posterior iliac crests exposed. After conventional iodine alcohol disinfection and local anesthesia with Lidocaine, a medullo-puncture needle was used to vertically penetrate the cortical bone into the bone marrow cavity. A heparin saline mixture (100 U/mL) of 5 mL in a 20 mL syringe was connected to the bone needle and 15 mL of bone marrow was extracted, for a total of 60 mL. After mixing well, and under strict aseptic conditions, 30 mL of the diluted bone marrow was added to 15 mL of Ficoll-Paque solution in every 50 mL centrifuge tube. Density-gradient centrifugation was performed according to the manufacturer’s instructions. Mononuclear cells were collected and washed twice in saline. If the children were crying, screaming, or moving in a restless motion to the extent that the surgeon could not proceed, chloral hydrate was used orally, or anally 1 h before the operation in a dosage according to the child’s weight (50 mg/kg) or by the body surface area (1.5 g/m^2^).

#### Preparation of human BMMSCs

BMMSCs were prepared as previously described [9]. Briefly, made from bone marrow MNC and containing 10% foetal bovine serum and Dulbecco’s modified Eagle’s medium, the cell suspension with 2 × 10^6^ to 4 × 10^6^/cm^2^ cells was seeded in culture flasks, and incubated at 37 °C in a humidified tissue culture incubator containing 5% CO2 and 95% air, with a change of culture medium every 3–5 days. After 10 days in culture, the adherent cells were trypsinised and passaged into a new culture bottle for further expansion. The BM-MSCs harvested from passage three were frozen before characterising MSC markers with flow cytometry. Flow cytometry results showed that ≥95% of cells expressed CD105, CD73, CD44, whereas the expression of CD45, CD34, CD31, CD146 and HLA-DR was 2% or less. One week before transplantation, the cryopreserved BM-MSCs were thawed for further culture and expansion. BM-MSCs between passages 4 and 6 were used for transplantation after characterising MSC markers with flow cytometry. Made after bone marrow MNC containing 10% foetal bovine serum and Dulbecco’s modified Eagle’s medium, the cell suspension with 2 × 10^6^ to 4 × 10^6^/cm^2^ cells was seeded in culture flasks.

### Cell transplantation

All of the patients were hospitalised and received four cell transplantations at an interval of 3–4 days. The protocol was performed as previously described [12]. In brief, a lumbar puncture was performed in the lumbar 3–4 or lumbar 4–5 intervertebral space. After the puncture needle had been confirmed to penetrate into the subarachnoid space, 2 mL of stem cell suspension was slowly injected into the space. Each time, the number of cells used was 1 × 10^6^/kg body weight.

### Intervention in control group

We used Bobath therapy (neuro-developmental treatment) as the unified method of treatment in the control group. The specific method is consistent with that of Knox Virginia et al. [13] including the following: (1) reflex inhibition (2) adjusting the key point (3) facilitating postural reflex to induce the maximal potential of the child without overexertion, form the movement posture of functional activity, and learn and comprehend the experience, and (4) percussion.

The patients in the control group received treatment in every 2 days, with a total course of 3 weeks. The Bobath method was administered by two skilled rehabilitation therapists.

### Assessment of efficacy

The gross motor function measure (GMFM) and fine motor function measure (FMFM) were used to evaluate the efficacy of cell therapy. All patients were evaluated at admission and followed-up at 3, 6 months, and 1 year after transplantation. All the evaluations were performed by two professional rehabilitation physicians who were blind to the study.

### Statistical analysis

All of the data are shown as the mean ± standard deviation (SD). Comparisons between before and after transplantation variables for each group were based on a paired *t* test. The GMFM and FMFM scores were compared among the three groups using the Student–Newman–Keuls test following one-way analysis of variance (ANOVA). A significant difference was indicated by P < 0.05. All statistical analyses were performed using SPSS (Version 16.0 for Windows, SPSS, Chicago, IL).

## Results

Two children in the BMMSC group and one child in the BMMNC group left the experiment due to their parent withdrawal. In all, there are 33 patients in the BMMSC group (18 boys and 15 girls), and 34 patients (18 boys and 16 girls) in the BMMNC group completed the experiment. The general details, as well as GMFM and FMFM scores are provided in Additional file 1, Tables 1 and 2.

### Baseline comparison

In comparison of the baseline of GMFM, FMFM, and dimensions among the BMMCS group, BMMNS group, and the control group, the differences were not statistically significant (P > 0.05).

#### Changes in gross motor function of children in the BMMSC group

Three months after cell transplantation, compared with the score before transplantation (Table 1), scores of A, B, and C dimensions of GMFM have significant improvement (P < 0.05 or P < 0.01). And, the total score of GMFM also improved significantly (P < 0.01). Scores of D and E dimensions also showed some improvement, but not significantly (P > 0.05). Six months after cell transplantation, compared with the score before transplantation, scores of A, B, C, and D dimensions of GMFM have significant improvement (P < 0.05 or P < 0.01). And, the total score of GMFM also improved significantly (P < 0.01). Scores of E dimensions have also shown some improvement, but not significantly (P > 0.05). Twelve months after cell transplantation, compared with the score before transplantation, scores of A, B, C, D and E dimensions and the total score of GMFM have significant improvement (P < 0.05 or P < 0.01).

**Table 1:** GMFM scores of the three groups after intervention $({\bar{\text{x}}} \pm {\text{s}})$

GMFM-domain	Baseline	3 months	6 months	12 months
BMMMSC group				
A	31.36 ± 7.24	36.70 ± 7.11**	38.39 ± 6.54**	39.73 ± 6.77**
B	31.97 ± 9.76	36.39 ± 9.65**	40.18 ± 8.60**	41.55 ± 8.60**
C	20.61 ± 3.55	23.39 ± 3.78*	24.21 ± 3.76*	25.30 ± 3.97**
D	5.21 ± 8.15	8.42 ± 9.59	9.58 ± 10.40*	10.18 ± 10.37*
E	6.06 ± 10.91	8.33 ± 11.64	9.64 ± 12.44	10.27 ± 12.39*
Total	95.21 ± 32.69	113.15 ± 34.93**	122 ± 35.50**	127.03 ± 35.80**^△^
BMMNC group				
A	31.67 ± 6.77	32.29 ± 6.72*	33.44 ± 6.67*	35.29 ± 6.54*
B	32.09 ± 9.65	33.12 ± 9.55*	34.38 ± 9.89*	36.29 ± 9.86*
C	20.65 ± 3.39	21.71 ± 3.52*	22.76 ± 3.41*	23.85 ± 3.55*
D	5.21 ± 7.99	5.71 ± 8.09	6.62 ± 8.08	8.09 ± 8.56
E	6.07 ± 9.99	6.65 ± 10.06	7.56 ± 10.26	8.38 ± 10.61
Total	95.68 ± 30.79	99.47 ± 30.89*	104.76 ± 31.39**	111.91 ± 31.68**
Control group				
A	31.46 ± 6.95	31.97 ± 6.89	32.49 ± 6.50	32.91 ± 6.18*
B	32.66 ± 9.22	33.2 ± 8.91	33.71 ± 8.80	34.34 ± 8.62*
C	20.71 ± 3.78	21.06 ± 3.93	21.66 ± 3.92	22.23 ± 4.02*
D	4.91 ± 7.13	5.26 ± 7.32	5.83 ± 7.37	6.37 ± 7.68
E	5.54 ± 9.13	5.86 ± 9.04	6.17 ± 9.08	6.66 ± 9.00
Total	95.26 ± 29.19	97.34 ± 28.96	99.86 ± 28.48*	102.51 ± 28.30**

** Compared with scores before transplantation/Bobath therapy, P < 0.01

* Compared with scores before transplantation/Bobath therapy, P < 0.05

^△^ Compared with scores 3 month after transplantation, P < 0.05

#### Changes in gross motor function of children in the BMMNC group

Three months after cell transplantation, compared with the score before transplantation (Table 1), scores of A, B, and C dimensions of GMFM have significant improvement (P < 0.05). And the total score of GMFM also improved significantly (P < 0.05). Scores of D and E dimensions have also shown some improvement, but not significantly (P > 0.05). Six months after cell transplantation, compared with the score before transplantation, scores of A, B, and C dimensions of GMFM have significant improvement (P < 0.05). And the total score of GMFM also improved significantly (P < 0.01). Scores of D and E dimensions have also shown improvement, but not significantly (P > 0.05). Twelve months after cell transplantation, compared with the score before transplantation, scores of A, B, and C dimensions and the total score of GMFM have significant improvement (P < 0.05 or P < 0.01). Scores of D and E dimensions had no significant improvement (P > 0.05).

#### Changes in gross motor function of children in the control group

Three and six months after rehabilitation therapy (Table 1), scores of all dimensions and the total of GMFM scores both have shown some improvement, but not significantly (P > 0.05). Twelve months after cell transplantation, compared with the score before transplantation, scores of A, B, and C dimensions and the total score of GMFM have significant improvement (P < 0.05 or P < 0.01). Scores of D and E dimensions had no significant improvement (P > 0.05).

#### Comparison of GMFM score among groups within each interval

Three months after each intervention, dimension A of the BMMSC group gained significant statistical difference compared with the other two groups (P < 0.05), whereas the difference of A dimension between the BMMNC group and the control is not significant (P > 0.05). Differences of dimension C between the BMMSC group and the BMMNC group, and the BMMNC group and the control group, are not significant (P > 0.05), whereas the difference between the BMMSC group and the control group is significant (P < 0.05); The differences of the B, D and E dimensions and GMFM total scores among the three groups are not significant (P > 0.05).

Six months after each intervention, dimensions A and B and the GMFM total scores of the BMMSC group gained significant statistical difference compared with the other two groups (P < 0.05), whereas the difference of A and B dimensions between the BMMNC group and the control is not significant (P > 0.05). The difference of dimension C between the BMMSC group and the BMMNC group, and the BMMNC group and the control group, is not significant (P > 0.05), whereas the difference of C dimension between the BMMSC group and the control group is significant (P < 0.05). The differences of D and E dimensions among the three groups are not significant (P > 0.05).

Twelve months after each intervention, dimension A, B, and C, GMFM total scores of the BMMSC group, gained significant statistical difference compared with the other two groups (P < 0.05), whereas the differences between the BMMNC group and the control are not significant (P > 0.05). The differences of D and E dimensions among the three groups are not significant (P > 0.05).

#### Changes in fine motor function of children in the BMMSC group

Three months after cell transplantation, compared with the score before transplantation (Table 2), scores of A, B, C, and D dimensions of FMFM in the BMMSC group patients have significant improvement (P < 0.05 or P < 0.01). And, the total score of FMFM also improved significantly (P < 0.01). Scores of E dimension have also gained some improvement, but not significantly (P > 0.05). Six months after cell transplantation, compared with the score before transplantation, scores of A, B, C, D and E dimensions and the total score of FMFM have significant improvement (P < 0.05 or P < 0.01). Twelve months after cell transplantation, compared with the score before transplantation, scores of A, B, C, D and E dimensions and the total score of FMFM have significant improvement (P < 0.05 or P < 0.01).

**Table 2:** FMFM scores of three groups after intervention $({\bar{\text{x}}} \pm {\text{s}})$

FMFM-domain	Baseline	3 months	6 months	12 months
BMMMSC group				
A	9.88 ± 2.81	12.03 ± 2.48**	15.00 ± 2.40**	17.58 ± 2.41**
B	10.15 ± 3.63	12.27 ± 3.28**	15.64 ± 3.36**	17.67 ± 3.49**
C	7.24 ± 4.19	10.55 ± 4.62**	13.70 ± 3.40**	15.58 ± 4.47**
D	6.36 ± 5.15	9.21 ± 6.20*	12.76 ± 7.37**	14.82 ± 7.10**
E	6.82 ± 6.47	8.88 ± 8.29	12.67 ± 8.82*	13.76 ± 8.76*
Total	40.45 ± 18.31	52.94 ± 20.94**	69.76 ± 21.67**^△^	79.39 ± 21.95**^△^
BMMNC group				
A	9.91 ± 2.85	10.47 ± 2.94*	11.15 ± 3.02*	11.94 ± 2.96*
B	10.14 ± 3.52	11.06 ± 3.35	12.18 ± 3.41*	12.68 ± 3.30*
C	7.24 ± 4.11	7.88 ± 4.04	8.53 ± 4.03*	9.38 ± 4.20*
D	6.47 ± 4.91	6.85 ± 5.03	7.88 ± 5.25*	8.94 ± 5.55*
E	6.79 ± 6.23	7.76 ± 6.80	8.65 ± 7.03*	9.65 ± 7.19*
Total	40.56 ± 17.57	44.03 ± 17.99	48.38 ± 18.47*	52.59 ± 18.89**
Control group				
A	9.83 ± 2.86	10.31 ± 2.54*	10.71 ± 2.67*	11.11 ± 2.89*
B	10.03 ± 3.68	10. 60 ± 3.99	11.03 ± 4.07	11.34 ± 4.01
C	7.26 ± 4.23	7.74 ± 4.56	7.94 ± 4.56	8.46 ± 4.13
D	6.46 ± 4.97	6.86 ± 4.95	7.26 ± 5.00	7.54 ± 5.12
E	6.86 ± 6.40	7.40 ± 6.63	7.83 ± 6.83	8.26 ± 6.96
Total	40.43 ± 15.88	42.91 ± 15.84	44.77 ± 16.27	46.71 ± 16.07*

** Compared with scores before transplantation/Bobath therapy, P < 0.01

* Compared with scores before transplantation/Bobath therapy, P < 0.05

^△^ Compared with scores 3 month after transplantation, P < 0.05

#### Changes in fine motor function of children in the BMMNC group

Three months after cell transplantation, compared with the score before transplantation (Table 2), the score of A dimension of FMFM in the BMMNC group patients has significant improvement (P < 0.05). Scores of B, C, D and E dimensions, as well as the FMFM total score, also gained some improvement, but not significantly (P > 0.05). Six months after cell transplantation, compared with the score before transplantation, scores of A, B, C, D, and E dimensions and the total score of FMFM have significant improvement (P < 0.05 or P < 0.01).Twelve months after cell transplantation, compared with the score before transplantation, scores of A, B, C, D, and E dimensions and the total score of FMFM have significant improvement (P < 0.05 or P < 0.01).

#### Changes in fine motor function of children in the control group

Three months after rehabilitation therapy, compared with the score before intervention (Table 2), the score of A dimension of FMFM in the control group patients has significantly improved (P < 0.05). Scores of B, C, D, and E dimensions, together with FMFM total score, have gained some improvement, but not significantly (P > 0.05). Six months after rehabilitation therapy, compared with the score before intervention, the score of A dimension has significant improvement (P < 0.05). Scores of B, C, D, and E dimensions, as well as FMFM total score, have received some improvement, but not significantly (P > 0.05).Twelve months after cell transplantation, scores of A and the total score of FMFM have significant improvement (P < 0.05).

#### Comparison of FMFM score among each group and each interval

Three months after each intervention, dimension A and C of the BMMSC group gained significant statistical difference compared with the other two groups (P < 0.05), whereas the difference of A and C dimensions between the BMMNC group and the control group is not significant (P > 0.05). The differences of B, D, and E dimensions and FMFM total scores among the three groups are not significant (P > 0.05).

Six months after each interventions, dimension A, B, C, D, and E and FMFM total scores of the BMMSC group gained significant statistical differences, compared with the other two groups (P < 0.05), whereas these differences between the BMMNC group and the control group are not significant (P > 0.05).

Twelve months after each intervention, dimension A, B, C, D, and E and FMFM total scores of the BMMSC group gained significant statistical differences, compared with the other two groups (P < 0.05), whereas these differences between the BMMNC group and the control group are not significant (P > 0.05).

### Adverse effects

The side effects of cell transplantation included the following: (1) fever: some patients experienced mild and moderate fever, which generally occurred on the first operative day and spontaneously recovered to normal levels, and the incidence was 8.8% (3/34) in the BMMNC group and 6.1% (2/33) in the BMMSC group; (2) low intracranial pressure reactions: the symptoms included nausea, vomiting, and headache. All of these symptoms were relieved or disappeared when the patients lay in bed in a supine position without a pillow and were treated with intravenous saline infusions. The incidence was 17.6% (6/34) in the BMMNC group and 12.1% (4/33) in the BMMSC group.

## Discussion

Spastic CP is a motor disorder with the feature of deteriorating motor nerve growth and permanent, irreversible limitation of activity. Due to the complexity of its pathogenesis and treatment,as well as its irreversibility,spastic CP has always been a challenge to CP patients, families, and the clinicians who treat them all over the world [14]. In recent years, along with the development of cell technology, a number of clinical studies have shown that stem cell transplantation in the treatment of traumatic brain injury [12], cerebral haemorrhage [15], stroke [16], spinal cord injury [17], ataxia [5, 18, 19] and many other kinds of diseases of the nervous system is safe and effective. These encouraging results open a new field for the treatment of CP. In addition, because of the plasticity of the childhood brain, this point in development also provides a great opportunity for the cell treatment of spastic CP [18]. There have been clinical studies using umbilical cord derived mesenchymal stem cells [11], embryonic stem cells [6], umbilical cord blood stem cells [20], bone marrow mesenchymal stem cells [9] and bone marrow mononuclear cells [4] for the treatment of spastic CP, and these treatments have achieved a certain effect. Among them, the BMMSCs and BMMNCs, owing to the ease of acquisition, no risk of rejection, and no ethical restrictions, have been widely used in the treatment of various neurological diseases. Because the two kinds of cells originate from the same source, and differ in the method of culturing, we compared and analysed the effect between autologous BMMSCs and autologous BMMNCs for the treatment of spastic CP, to guide future treatment.

This study concludes, in terms of gross motor function, 3 months after transplantation, the BMMSC group and the BMMNC group begin to gain significant improvements (A, B, and C dimensions and GMFM total scores). These findings are the same as those for previous studies (Alok Sharma et al. [4] using BMMNCs; Guojun Chen et al. using neural stem cell-like cells derived from autologous BMMSCs) about the timing of outcomes [14]. Six months after the intervention, A and B dimensions and GMFM total scores in the BMMSC group were better than those for the other two groups, whereas the differences between the BMMNC group and the control group are not significant. Twelve months after the intervention, A, B, and C dimensions and GMFM total scores in the BMMSC group were better than those for the other two groups. The results indicate that, 3 months after transplantation, in gross motor skills, BMMSC and BMMNC could significantly improve the gross motor function of children with spastic CP, and the outcomes are better than those for the rehabilitation group. However, with the extension of time, improvement of the gross motor function of the BMMSC group, especially in A, B, and C dimensions and the total score of GMFM, is more persistent than that of the BMMNC group. Six and twelve months after transplantation, there is no difference between the BMMNC group and the rehabilitation group, which also indicates that BMMNCs have a poor persistence in improving gross motor function. In terms of fine motor function, we also found that 6 months after treatment, considering each dimensions and total score of FMFM, the BMMSC group was better than the BMMNC and the rehabilitation treatment group, which further proved that the continuity of improvement of BMMSC for the motor function of children with spastic CP is better than BMMNC.

As for the insignificant improvement of D and E dimensions, several possibilities may have caused this phenomenon: (1) From the A to E dimension, the corresponding action of muscle strength and coordination required to complete the task is gradually increasing; thus, the children’s improvement from A dimension gradually expanded to E dimension. (2) Because many patients were infants or young children, and are thus not quite capable of completing the tasks in the D and E dimensions, the resulting D and E scores showed no significant difference before and after transplantation. The related research needs to further confirm our results.

Most of the treatment mechanisms of cell transplantation were studied through animal experiment, and there are three major mechanisms: (1) regeneration and differentiation mechanism: the transplanted cells differentiate into corresponding cells such as neurons, oligodendrocytes or astrocytes by homing to the damaged area [21, 22]; (2) paracrine mechanism: the transplanted cells can secrete a variety of cytokines, such as neurotrophic factors, anti-inflammatory cytokines, and angiogenic factors. BMMSCs have stronger ability of secretion than do BMMNCs [23, 24]; and (3) Immune regulation mechanism: mesenchymal stem cells (MSCs) can regulate the body’s immune system and inhibit the abnormal immune response [25, 26]. We believe that the major treatment mechanism of the transplanted stem cells are the paracrine mechanism [27, 28] and the vascular regeneration mechanism [24], especially in that human MSC subpopulations express a variety of neuro-regulatory molecules and promote neuronal cell survival and neurogenesis [29]. In addition, studies showed that BMMSCs are better than BMMNCs in both the capacity and the ability of paracrine and differentiation [23]. Many studies obtained the same conclusion in different types of disease treated with BMMSCs and BMMNCs. Lu D demonstrated that BMMSC therapy may be better tolerated and more effective than BMMNCs for increasing lower limb perfusion and promoting foot ulcer healing in diabetic patients with critical limb ischemia [30]. The Mazo M study also showed that MSC provides a long-term superior benefit over whole BMMNC transplantation in a rat model of chronic myocardial infarction [31]. These mechanisms partly explain why BMMSC has a better therapeutic effect than BMMNC in children with spastic CP.

Many clinical studies reported that the therapeutic effect of BMMSC and BMMNC is not associated with serious complications [4, 20]. In this study, no children in either of the two cell transplantation groups had any serious adverse reactions; some did experience low fever and intracranial pressure, which is the same as found in other research studies. We found that patients treated with BMMSCs and BMMNCs had no serious adverse reactions. Two patients (2/33) in the BMMSC group and three patients (3/34) in the BMMNC group had a low fever, while the number in BMMNC group is three (3/34). Low intracranial pressure symptoms are associated with lumbar surgery, and are not associated with cell transplantation.

## Conclusions

Through this research, we further confirmed that BM-MSCs transplantation for the treatment of CP is safe and feasible, and can improve gross and fine motor function significantly in children with spastic CP. And, compared with the results of BMMNC treatment, BMMSC treatment significantly improved the gross and fine motor function of children with spastic CP children improved significantly in terms of gross motor or the fine motor functions.

## Additional file

**Additional file 1: Table S1.** Clinical characteristics of the 33 patients in the BMMSC group. **Table S2.** Clinical characteristics of the 34 patients in the BMMNC group. **Table S3.** Clinical characteristics of the 35 patients in the control group.

## Abbreviations

MSCs: mesenchymal stem/stromal cells
BMMSCs: bone marrow mesenchymal stem cells
UCMSCs: umbilical cord-derived mesenchymal stem cells
BMMNCs: bone marrow mononuclear cells
GMFCS: gross motor function classification system
GMFM: gross motor function measure
FMFM: fine motor function measure
CP: cerebral palsy
