# Supplementary material for: Comparative analysis of curative effect of bone marrow mesenchymal stem cell and bone marrow mononuclear cell transplantation for spastic cerebral palsy
Source: J Transl Med. 2017 Feb 24;15:48. doi: 10.1186/s12967-017-1149-0 (PMC5324263; doi:10.1186/s12967-017-1149-0)
Supplement: Supplementary file 1 — Additional file 1: Table S1. Clinical characteristics of the 33 patients in the BMMSC group. Table S2. Clinical characteristics of the 34 patients in the BMMNC group. Table S3. Clinical characteristics of the 35 patients in the control group. [file 12967_2017_1149_MOESM1_ESM.docx]

Baseline characteristics of the patients

**Supplementary Table 1 Clinical characteristics of the 33 patients in the**

**BMMSC group.**

| **Case no.** | **Gender** | **Age（M）** | **GMFCS**  **level** | **Case no.** | **Gender** | **Age（M）** | **GMFCS**  **level** |
| --- | --- | --- | --- | --- | --- | --- | --- |
| 1 | M | 36 | Ⅲ | 18 | F | 36 | Ⅳ |
| 2 | M | 36 | Ⅳ | 19 | M | 48 | Ⅱ |
| 3 | F | 7 | Ⅲ | 20 | F | 108 | Ⅴ |
| 4 | F | 24 | Ⅳ | 21 | M | 19 | Ⅱ |
| 5 | M | 36 | Ⅱ | 22 | F | 72 | Ⅳ |
| 6 | F | 10 | Ⅲ | 23 | F | 48 | Ⅳ |
| 7 | M | 60 | Ⅲ | 24 | F | 48 | Ⅴ |
| 8 | F | 24 | Ⅲ | 25 | F | 36 | Ⅴ |
| 9 | F | 72 | Ⅱ | 26 | M | 96 | Ⅳ |
| 10 | F | 48 | Ⅲ | 27 | F | 96 | Ⅱ |
| 11 | M | 96 | Ⅲ | 28 | M | 36 | Ⅴ |
| 12 | M | 36 | Ⅲ | 29 | F | 18 | Ⅴ |
| 13 | F | 24 | Ⅱ | 30 | M | 96 | Ⅳ |
| 14 | F | 36 | Ⅳ | 31 | F | 36 | Ⅳ |
| 15 | M | 132 | Ⅴ | 32 | M | 60 | Ⅲ |
| 16 | M | 60 | Ⅲ | 33 | M | 48 | Ⅲ |
| 17 | F | 36 | Ⅳ |  |  |  |  |

**Supplementary Table 2 Clinical characteristics of the 34 patients in the**

**BMMNC group.**

| **Case no.** | **Gender** | **Age（M）** | **GMFCS**  **level** | **Case no.** | **Gender** | **Age（M）** | **GMFCS**  **level** |
| --- | --- | --- | --- | --- | --- | --- | --- |
| 1 | F | 96 | Ⅲ | 20 | F | 50 | Ⅱ |
| 2 | M | 15 | Ⅱ | 21 | M | 37 | Ⅳ |
| 3 | F | 24 | Ⅲ | 22 | F | 40 | Ⅳ |
| 4 | F | 48 | Ⅳ | 23 | M | 50 | Ⅱ |
| 5 | M | 36 | Ⅱ | 24 | F | 92 | Ⅴ |
| 6 | F | 36 | Ⅴ | 25 | F | 36 | Ⅳ |
| 7 | M | 50 | Ⅲ | 26 | F | 36 | Ⅲ |
| 8 | F | 58 | Ⅳ | 27 | F | 120 | Ⅴ |
| 9 | M | 35 | Ⅲ | 28 | M | 96 | Ⅱ |
| 10 | F | 120 | Ⅳ | 29 | F | 72 | Ⅳ |
| 11 | M | 18 | Ⅴ | 30 | M | 60 | Ⅲ |
| 12 | M | 25 | Ⅲ | 31 | F | 50 | Ⅴ |
| 13 | F | 17 | Ⅴ | 32 | M | 24 | Ⅲ |
| 14 | F | 50 | Ⅱ | 33 | F | 10 | Ⅳ |
| 15 | M | 36 | Ⅴ | 34 | M | 36 | Ⅱ |
| 16 | M | 50 | Ⅲ |  |  |  |  |
| 17 | F | 30 | Ⅲ |  |  |  |  |
| 18 | M | 96 | Ⅱ |  |  |  |  |
| 19 | M | 96 | Ⅱ |  |  |  |  |

**Supplementary Table 3 Clinical characteristics of the 35 patients in the**

**control group.**

| **Case no.** | **Gender** | **Age（M）** | **GMFCS**  **level** | **Case no.** | **Gender** | **Age（M）** | **GMFCS**  **level** |
| --- | --- | --- | --- | --- | --- | --- | --- |
| 1 | F | 84 | Ⅲ | 20 | F | 48 | Ⅱ |
| 2 | M | 12 | Ⅱ | 21 | M | 37 | Ⅳ |
| 3 | F | 24 | Ⅲ | 22 | F | 40 | Ⅳ |
| 4 | F | 50 | Ⅳ | 23 | M | 50 | Ⅱ |
| 5 | M | 39 | Ⅱ | 24 | F | 92 | Ⅴ |
| 6 | F | 36 | Ⅴ | 25 | F | 36 | Ⅳ |
| 7 | M | 54 | Ⅲ | 26 | F | 36 | Ⅲ |
| 8 | F | 58 | Ⅳ | 27 | F | 120 | Ⅴ |
| 9 | M | 36 | Ⅲ | 28 | M | 96 | Ⅱ |
| 10 | F | 120 | Ⅳ | 29 | F | 72 | Ⅳ |
| 11 | M | 18 | Ⅴ | 30 | M | 60 | Ⅲ |
| 12 | M | 25 | Ⅲ | 31 | F | 50 | Ⅴ |
| 13 | F | 17 | Ⅴ | 32 | M | 24 | Ⅲ |
| 14 | F | 50 | Ⅱ | 33 | F | 10 | Ⅳ |
| 15 | M | 36 | Ⅴ | 34 | M | 36 | Ⅱ |
| 16 | M | 50 | Ⅲ | 35 | F | 24 | Ⅲ |
| 17 | F | 30 | Ⅲ |  |  |  |  |
| 18 | M | 96 | Ⅱ |  |  |  |  |
| 19 | M | 96 | Ⅱ |  |  |  |  |
